# ^18^F-FDG PET/CT-based radiomics nomogram could predict bone marrow involvement in pediatric neuroblastoma

**DOI:** 10.1186/s13244-022-01283-8

**Published:** 2022-09-04

**Authors:** Lijuan Feng, Xu Yang, Xia Lu, Ying Kan, Chao Wang, Dehui Sun, Hui Zhang, Wei Wang, Jigang Yang

**Affiliations:** 1 Department of Nuclear Medicine, Beijing Friendship Hospital, Capital Medical University, 95 Yong An Road, Xi Cheng District, Beijing, 100050 China; 2 Sinounion Medical Technology (Beijing) Co., Ltd., Beijing, 100192 China; 3 Department of Biomedical Engineering, School of Medicine, Tsinghua University, Beijing, 100084 China

**Keywords:** Neuroblastoma, Positron emission tomography/computed tomography, Radiomics, Nomogram

## Abstract

**Objective:**

To develop and validate an ^18^F-fluorodeoxyglucose (FDG) positron emission tomography/computed tomography (PET/CT)-based radiomics nomogram for non-invasively prediction of bone marrow involvement (BMI) in pediatric neuroblastoma.

**Methods:**

A total of 133 patients with neuroblastoma were retrospectively included and randomized into the training set (*n* = 93) and test set (*n* = 40). Radiomics features were extracted from both CT and PET images. The radiomics signature was developed. Independent clinical risk factors were identified using the univariate and multivariate logistic regression analyses to construct the clinical model. The clinical-radiomics model, which integrated the radiomics signature and the independent clinical risk factors, was constructed using multivariate logistic regression analysis and finally presented as a radiomics nomogram. The predictive performance of the clinical-radiomics model was evaluated by receiver operating characteristic curves, calibration curves and decision curve analysis (DCA).

**Results:**

Twenty-five radiomics features were selected to construct the radiomics signature. Age at diagnosis, neuron-specific enolase and vanillylmandelic acid were identified as independent predictors to establish the clinical model. In the training set, the clinical-radiomics model outperformed the radiomics model or clinical model (AUC: 0.924 vs. 0.900, 0.875) in predicting the BMI, which was then confirmed in the test set (AUC: 0.925 vs. 0.893, 0.910). The calibration curve and DCA demonstrated that the radiomics nomogram had a good consistency and clinical utility.

**Conclusion:**

The ^18^F-FDG PET/CT-based radiomics nomogram which incorporates radiomics signature and independent clinical risk factors could non-invasively predict BMI in pediatric neuroblastoma.

**Supplementary Information:**

The online version contains supplementary material available at 10.1186/s13244-022-01283-8.

## Key points

- Radiomics signature is valuable in the non-invasively diagnosis of bone marrow involvement.
- Age, NSE and VMA are the independent predictors of bone marrow involvement
- The nomogram incorporating radiomics signature and independent clinical risk factors improves predictive performance.

## Introduction

Neuroblastoma is a malignant neuroectodermal tumor that originated from cells of the neural crest and is the most common extracranial solid malignant tumor of childhood [1, 2]. About 50% of neuroblastoma patients had metastasis at the time of initial diagnosis, which is frequently associated with unsatisfactory outcomes. The bone marrow is the most common site of metastasis in neuroblastoma [3], and the spread of tumor cells to bone marrow is a grim prognostic factor in neuroblastoma patients [4]. The prognosis is significantly better in those without bone marrow involvement (BMI) than in those with BMI [5, 6]. Moreover, BMI is one of the most important diagnostic criteria and an important risk factor for staging according to the International Neuroblastoma Staging System (INSS) and the International Neuroblastoma Risk Group staging system [7–9]. Therefore, establishing an early predictive model for BMI in neuroblastoma patients is crucial for prognosis and the decision of treatment strategy.

According to the recommendations of the INSS, the cytology of aspirates and histology of biopsies are the gold standard with which to assess BMI in patients with neuroblastoma [5, 7, 8]. Considering that most neuroblastoma patients are children, the bone marrow aspirates and biopsies must be performed only by experienced health care providers who have been well-trained in the technique [10]. However, as invasive methods, the aspirates or biopsies may cause adverse events such as hemorrhage, infection and persistent pain. Moreover, bone marrow aspirates or biopsies had higher healthcare costs for the method, including pathology, sedation, anesthesia and surgical suite time [11]. Because of these limitations, better non-invasive computational tools should be developed that can effectively identify neuroblastoma patients with BMI.

Radiomics analysis converts medical images into mineable high-dimensional data by extracting innumerable quantitative features with high-throughput computing [12]. Once the high-dimension feature data describing quantitative attributes of volumes of interest is available, artificial intelligence, machine learning, or statistical approaches can be used to build classifier or regression modeling for disease detection, diagnosis, evaluation of prognosis and prediction of treatment response.

In recent years, ^18^F-fluorodeoxyglucose (FDG) positron emission tomography/computed tomography (PET/CT) has been used for the evaluation of BMI and is very helpful in evaluating the BMI in many malignancies [13, 14]. ^18^F-FDG PET/CT-based texture image features may provide predictive and prognostic biomarkers which performed better than standardized uptake value parameters, metabolic tumor volume and total lesion glycolysis in lung cancer [15]. Although ^18^F-FDG PET/CT texture analyses have been applied as a new method for differentiating BMI in lymphoma [16], there has been no study about radiomics based on ^18^F-FDG PET/CT used for the prediction of BMI in pediatric neuroblastoma. Therefore, the present study aimed to develop and validate a radiomics nomogram that integrated the radiomics signature and the independent clinical risk factors for non-invasively prediction of BMI in pediatric patients with neuroblastoma.

## Materials and methods

### Patients

This retrospective study was approved by the institutional review board and waived the requirement for written informed consent.

**Fig. 1:**
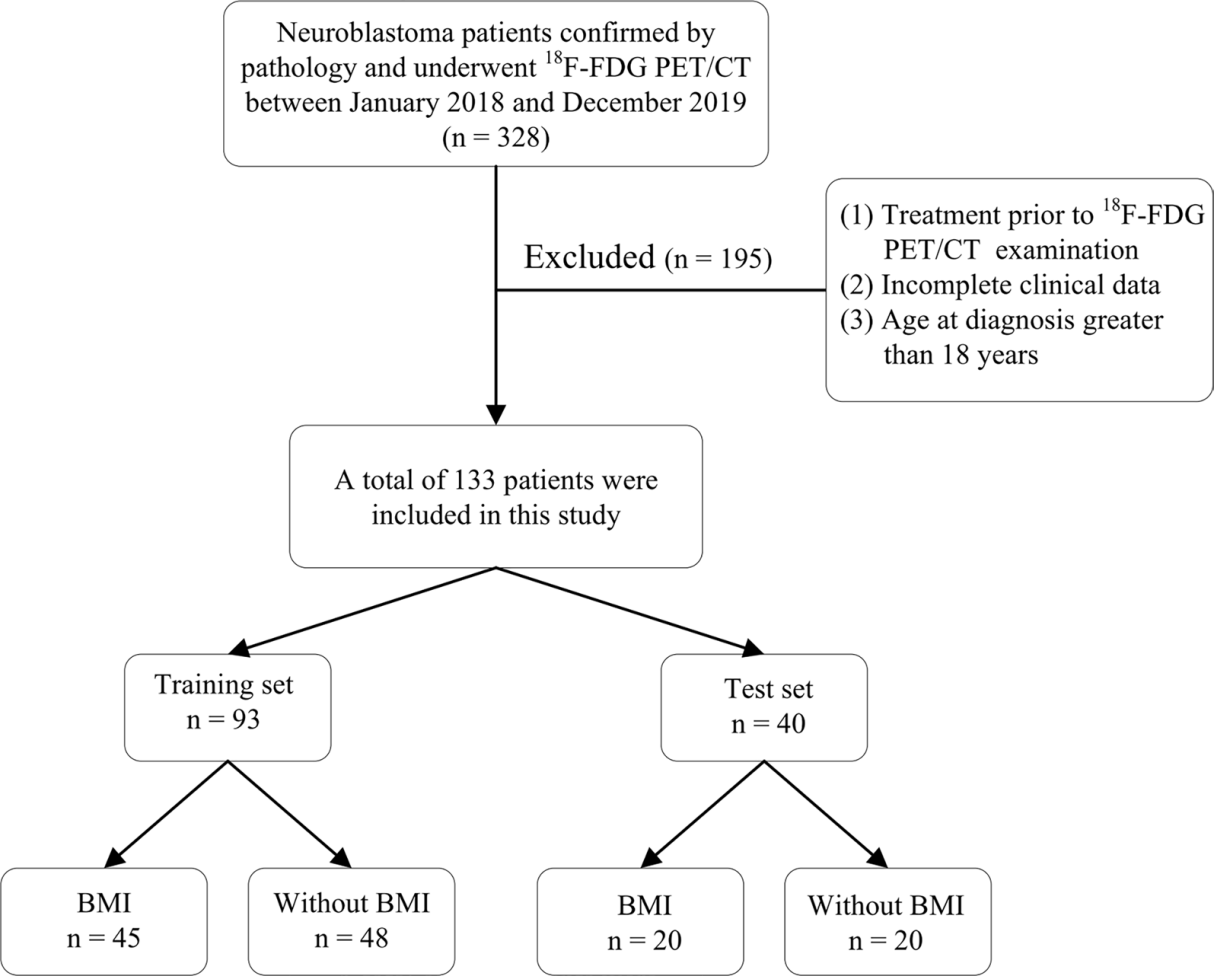
The flow chart for patient selection

We retrospectively included a cohort of 328 patients who underwent ^18^F-FDG PET/CT in our institution with pathologically confirmed neuroblastoma between January 2018 and December 2019. The inclusion criteria consisted of (1) patients with neuroblastoma who underwent bone marrow aspirates or biopsies and were assessed BMI using morphologic criteria in conjunction with appropriate antibodies; (2) ^18^F-FDG PET/CT scan performed within 30 days before the bone marrow aspirates or biopsies. The exclusion criteria included the following: (1) patients who received tumor-related treatments such as chemotherapy, radiotherapy and surgical excision prior to ^18^F-FDG PET/CT examination; (2) patients with incomplete clinical data; (3) patients were greater than 18 years of age at diagnosis. Finally, a total of 133 patients (58 males and 75 females; median age, 3.2 years; range, 1.7–4.7 years) were retrospectively included in this study. According to the result of bone marrow aspirates or biopsies, there were 65 patients with BMI and 68 patients without BMI. The flow chart for patient selection is shown in Fig. 1.

Stratified sampling according to the BMI stratification was implemented to balance the positive and negative cases, and the final 133 cases were randomly divided into the training set and test set according to a ratio of 7:3, which resulted in 93 cases being divided into the training set and 40 cases being divided into the test set.

The baseline data of each patient were obtained by reviewing the medical records, which included the following aspects: (1) clinical information, (2) laboratory indicators, (3) PET metabolic parameters, and (4) pathological-related information. We defined these data as clinical characteristics. Laboratory indicators including neuron-specific enolase (NSE), serum ferritin, lactate dehydrogenase (LDH), urine vanillylmandelic acid (VMA) and homovanillic acid (HVA) were acquired within two weeks before therapy.

### PET/CT imaging acquisition

All patients in the cohort underwent whole body ^18^F-FDG PET/CT (Biograph mCT-64 PET/CT; Siemens, Knoxville) scans according to European Association of Nuclear Medicine guidelines for tumor imaging [17, 18]. Patients were instructed to ban from intense exercises for at least 24 h before PET/CT scan and fast at least 6 h before ^18^F-FDG injection. A mean dose of 3 mCi (mean 0.14 mCi/kg) was administrated considering the patients are children. A low-dose CT scan (CT scanning parameters: tube voltage 120 keV, thickness 2 mm, matrix size 512 × 512) for viewing anatomic structures and attenuation correction was performed an hour after the injection. PET scan with three-dimension image mode and 2 min per bed setting followed immediately after CT acquisition. PET images were reconstructed with the time-of-flight ordered subsets-expectation maximization algorithm. All corrections for quantitative interpretation, including detector sensitivity normalization, dead time, random, scatter, attenuation and decay correction were applied during reconstruction. A Gaussian smoothing filter with a full width at half-maximum of 5 mm was applied to the PET images. The PET images’ parameters were as follows: pixel size 4.07 mm × 4.07 mm, 3 mm slice thickness, and matrix size 200 × 200.

### Tumor segmentation, radiomics features extraction and selection

**Fig. 2:**
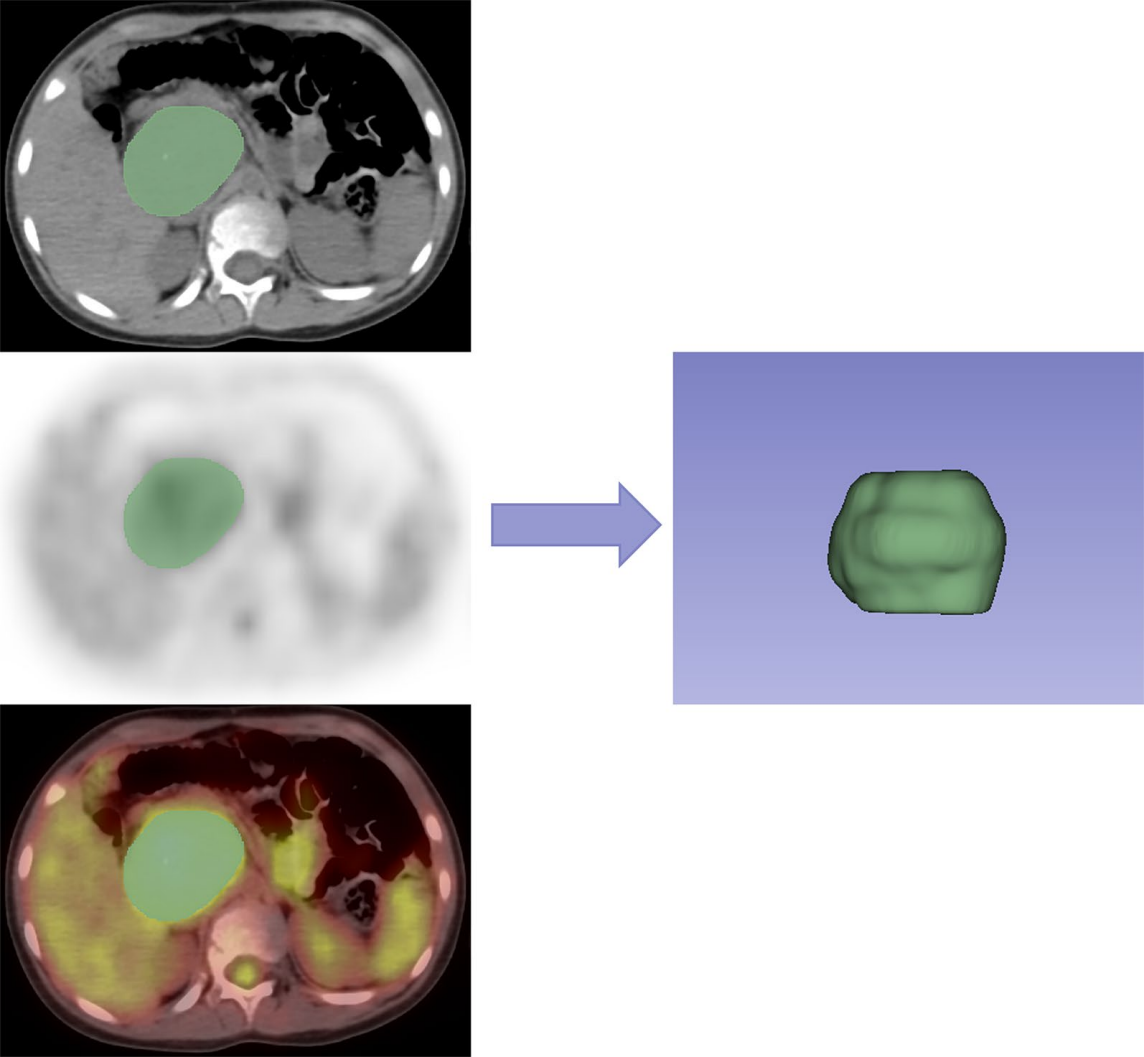
Schematic representation of the tumor segmentation by 3D Slicer

Image segmentation was performed semi-automatically with a commonly used open-source software (3D Slicer, Version 4.10.1) by reader 1 (W.W. with 7 years of experience in pediatric oncologic radiology). An example of ROI segmentation is shown in Fig. 2. The intraclass correlation coefficient (ICC) was used to assess the reproducibility of the selected features. A total of 30 cases (15 with BMI and 15 without BMI) of CT images and PET images randomly selected from the whole cohort were independently performed repeat segmentation by reader 1 and reader 2 (Y.K. with 10 years of experience in pediatric oncologic radiology). The readers were blinded to the clinical information when performing the segmentation.

Radiomics features were extracted from both CT and PET images using Pyradiomics in Python (version 3.7.8), an open-source python package for the extraction of radiomics features from medical imaging. A fixed bin width (0.3 standardized uptake value for PET image and 25 Hounsfield Units [HUs] for CT image) had been chosen to discretize gray value discretization for texture features extraction [19, 20]. Furthermore, filters including wavelet, square, and logarithm et al. were applied to the original CT and PET images for calculating high-dimensional features.

The features with ICC > 0.8 were considered reliable and maintained for subsequent analysis [21–23]. Then, the Pearson’s correction coefficients and Spearman’s rank correlation coefficient were calculated to examine redundant and collinear features, and features with mutual correlation coefficients > 0.9 were removed [24]. Finally, the least absolute shrinkage and selection operator (LASSO) regression with fivefold cross-validation was applied for feature selection.

### Radiomics model, clinical model and radiomics nomogram construction and evaluation

Using the most optimal features to construct the radiomics signature. The radiomics score (Rad score) was calculated for each patient via the combination of the selected features with their respective weight coefficients.

The univariate logistic regression analysis was used to assess the difference in clinical characteristics between BMI and without BMI in the training set. Then, variables with *p* < 0.05 in the univariate logistic regression analysis were applied to multivariate logistic regression analysis to elucidate the independent clinical risk factors. Meanwhile, multivariate logistic regression analysis was applied to build the clinical model was built based on the independent clinical risk factors.

The clinical-radiomics model, which incorporated the independent clinical risk factors and Rad score, was constructed using multivariable logistic regression analysis and finally presented as a radiomics nomogram in the training set. Logistic regression is a classical statistical model that internally has a linear regression, which is topped up by a sigmoid function such that the output of the model is a probability estimate between 0 and 1 [25].

The predictive performance of the clinical-radiomics model was evaluated by receiver operating characteristic (ROC) curves, calibration curves, and decision curve analysis (DCA). A calibration curve, obtained by plotting the actual probability against the nomogram-predicted probability, was used to evaluate the calibration of the nomogram. DCA was employed to evaluate the clinical utility of the radiomics nomogram.

Moreover, we applied two other machine learning methods, naive bayes and neural network, to build clinical-radiomics models, and then compared the predictive performance of the models obtained by each machine learning method based on such matrices as the area under the curve (AUC), accuracy, precision, F1-score and recall.

### Statistical analysis

Categorical variables were expressed as counts (n) and percentages (%), while continuous variables were expressed as mean values ± standard deviation or medians with the interquartile ranges. Statistical analyses were performed using R (version 4.0.3) and IMB SPSS Statistics (version 26.0). Two-sided with *p* < 0.05 was considered statistically significant. Univariate analysis was used to compare differences in the clinical factors between the training and test sets, using the independent t-test or Mann–Whitney U test for quantitative data, and the chi-squared test for categorical variables. Clinical independent predictors were screened using univariate and multivariate logistic regression analysis. The DeLong test was used to compare the AUC values of different models. The nomogram and calibration curve were depicted using the “rms (R)” package. DCA was performed using the “rmda (R)” package.

## Results

### Patient characteristics

The clinical characteristics of the patients in the training and test sets were summarized in Table 1. Univariate analysis indicated that there were no significant differences in all of these clinical factors between training and test sets.The complete dataset is available in Additional file 1.

**Table 1:** Characteristics of patients with neuroblastoma in the training set and test set

Characteristics	All Patients (*n* = 133)	Training set (*n* = 93)	Test set (*n* = 40)	*p* value
Age at diagnosis (years)	3.2 (1.7–4.7)	2.8 (1.4–4.7)	3.4 (2.0–4.7)	0.520
Gender				0.866
Female	75 (54.7%)	52 (54.8%)	23 (54.5%)	
Male	58 (45.3%)	41 (45.2%)	17 (45.5%)	
BMI				0.865
Yes	65 (48.9%)	45 (48.4%)	20 (50.0%)	
No	68 (51.1%)	48 (51.6%)	20 (50.0%)	
Maximum diameter(cm)	9.5 ± 3.9	9.3 ± 3.6	10.0 ± 4.6	0.373
MYCN Status				0.845
Amplified	22 (17.3%)	15 (16.7%)	7 (18.2%)	
Not Amplified	111 (82.7%)	78 (83.3%)	33 (81.8%)	
11q Aberration				0.575
Yes	55 (41.0%)	37 (45.2%)	18 (34.5%)	
No	78 (59.0%)	56 (54.8%)	22 (65.5%)	
1p Aberration				0.598
Yes	52 (41.0%)	35 (41.7%)	17 (40.0%)	
No	81 (59.0%)	58 (58.3%)	23 (60.0%)	
INSS Stage				0.435
1, 2, 3, 4S	43 (30.9%)	32 (31.0%)	11 (30.9%)	
4	90 (69.1%)	61 (69.0%)	29 (69.1%)	
COG Risk Stratification				0.540
Low, Intermediate	45 (32.4%)	33 (31.0%)	12 (34.5%)	
High	88 (67.6%)	60 (69.0%)	28 (65.5%)	
NSE (ng/mL)	237.5 (64.5–631.5)	217.9 (61.7–532.3)	315.3 (72.9–798.6)	0.290
Ferritin (ng/mL)	214.5 (72.8–295.8)	232.4 (91.2–303.5)	153.6 (64.6–288.6)	0.421
LDH (U/L)	578.0 (339.5–1038.0)	567.0 (339.5–904.5)	656.5 (342.0–1184.5)	0.589
VMA (μmol/L)	162.5 (46.2–501.8)	162.5 (49.8–552.6)	162.5 (32.2–473.3)	0.937
HVA (μmol/L)	36.4 (14.2–92.3)	36.4 (13.8–91.4)	36.4 (20.4–186.1)	0.595
SUVmax	5.4 (4.0–7.8)	5.2 (4.0–8.6)	5.8 (4.0–7.6)	0.941
SUVmean	2.0 (1.6–2.6)	2.0 (1.6–2.6)	2.2 (1.6–2.6)	0.669
MTV (mL)	130.3 (52.5–292.5)	130.3 (52.4–266.5)	131.8 (54.4–364.6)	0.772
TLG	269.5 (95.5–651.4)	248.0 (96.7–524.1)	296.8 (86.2–854.0)	0.662

*BMI* Bone marrow involvement, *COG* Children's Oncology Group, *HVA* Homovanillic acid, *INSS* International Neuroblastoma Staging System, *LDH* Serum lactate dehydrogenase, *MTV* Metabolic tumor volume, *NSE* Neuron-specific enolase, *TLG* Total lesion glycolysis, *VMA* Vanillylmandelic acid

### Feature selection and radiomics signature development

**Fig. 3:**
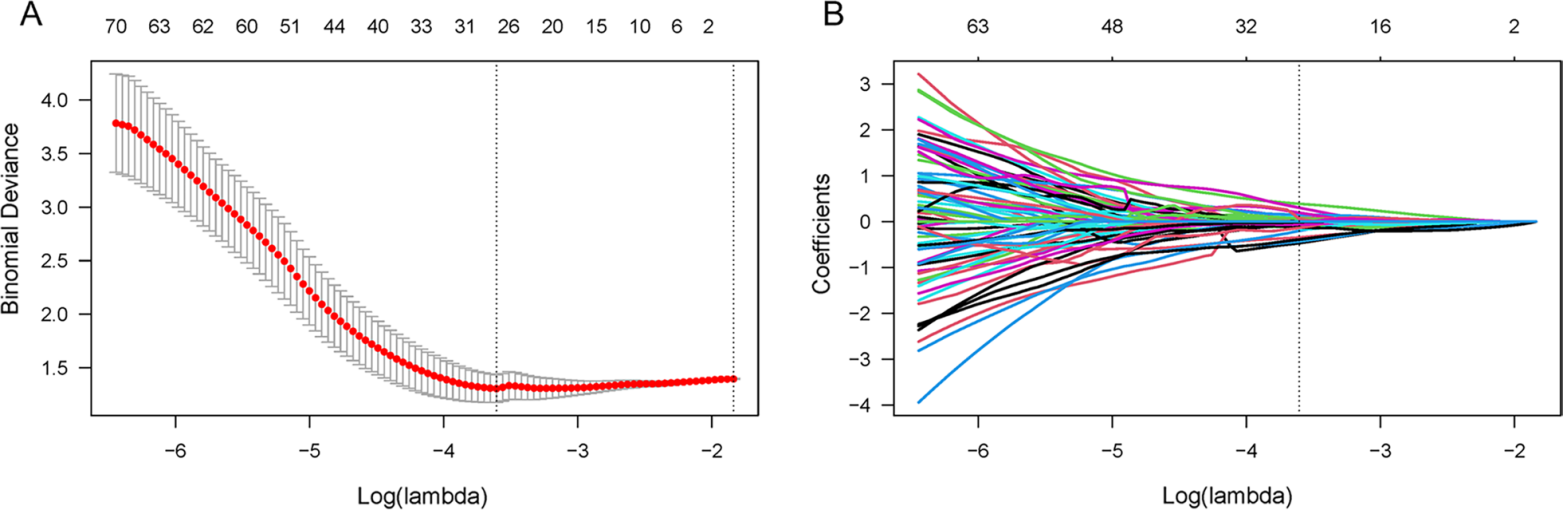
Radiomics feature selection using the least absolute shrinkage and selection operator (LASSO) regression. **A** The tuning parameter lambda (λ) in the LASSO regression model was selected via five-fold cross-validation based on minimum criteria. The LASSO regression model shows the best predictive performance when the λ value was set as 0.027167 and log(λ) was − 3.605761, at which point 25 features were selected. **B** The dotted vertical line was plotted at the selected λ value, resulting in 25 non-zero-coefficient features

**Fig. 4:**
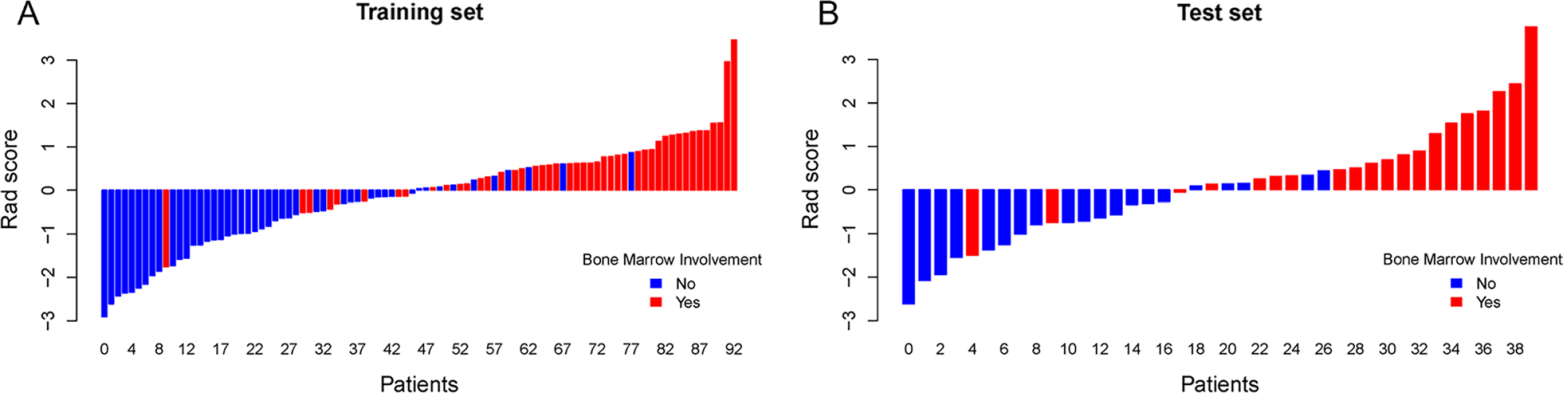
**A** Rad score of each patient in the training set. **B** Rad score of each patient in the test set

A total of 2632 radiomics features were extracted and the definition complies with Imaging Biomarker Standardization Initiative [26]. After assessing the robustness, 1016 out of 2632 features were retained for model building, with ICC > 0.8. One hundred and seventy-one features were identified as independent after Pearson’s correlation and Spearman’s rank correlation analysis. Eventually, twenty-five predictive radiomics features were chosen to generate the radiomics signature by LASSO regression. Figure 3 showed the selection of radiomics features using the LASSO regression. The detail of the 25 radiomics features selected by LASSO (Additional file 2: Fig. 1) and the formula for the Rad score were described in the Additional file. The Rad score was statistically significant in the training set (*p* < 0.001) and test set (*p* < 0.001) between with BMI and without BMI patients. Figure 4 showed the Rad score for each patient, and most patients with BMI had a higher Rad score than those without BMI. It indicated that the Rad score can be a good differentiator for neuroblastoma patients with or without BMI.

### Construction of the clinical model and radiomics nomogram

Among all patients’ clinical characteristics which excluded pathological-related information, nine significant predictors including age at diagnosis, gender, maximum diameter, NSE, ferritin, LDH, VMA, metabolic tumor volume and total lesion glycolysis were identified by univariate regression analysis. Age at diagnosis, NSE and VMA were identified as independent predictors of BMI by multivariate logistic regression analysis (Table 2). The AUC of the clinical model constructed by the three independent predictors of BMI was 0.875 (95% confidence interval [CI], 0.806–0.944) and 0.910 (95% CI, 0.821–0.999) in the training and test sets, respectively.

**Table 2:** Univariate and multivariate logistic regression analysis of clinical characteristics for predicting the BMI in the training set

Characteristics	Univariate		Multivariate	
	OR (95% CI)	*p* value	OR (95% CI)	*p* value
Age at diagnosis (years)	1.547 (1.219, 1.962)	< 0.001	1.551 (1.184, 2.031)	0.001
Gender	2.500 (1.079, 5.792)	0.033	NA	NA
Maximum diameter(cm)	1.215 (1.065, 1.387)	0.004	NA	NA
NSE (ng/mL)	1.003 (1.002, 1.005)	< 0.001	1.003 (1.002, 1.005)	< 0.001
Ferritin (ng/mL)	1.007 (1.003, 1.011)	< 0.001	NA	NA
LDH (U/L)	1.000 (1.000, 1.001)	0.047	NA	NA
VMA (μmol/L)	1.002 (1.000, 1.003)	0.010	1.002 (1.001, 1.003)	0.006
HVA (μmol/L)	1.003 (0.999, 1.006)	0.161	NA	NA
SUVmax	1.309 (0.860, 1.991)	0.403	NA	NA
SUVmean	1.309 (0.860, 1.991)	0.209	NA	NA
MTV (mL)	1.003 (1.000, 1.006)	0.032	NA	NA
TLG	1.001 (1.000, 1.002)	0.029	NA	NA

*CI* Confidence interval, *HVA* Homovanillic acid, *LDH* Serum lactate dehydrogenase, *MTV* Metabolic tumor volume, *NA* Not available, *NSE* Neuron-specific enolase, *OR* Odds ratio, *TLG* Total lesion glycolysis, *VMA* Vanillylmandelic acid

**Fig. 5:**
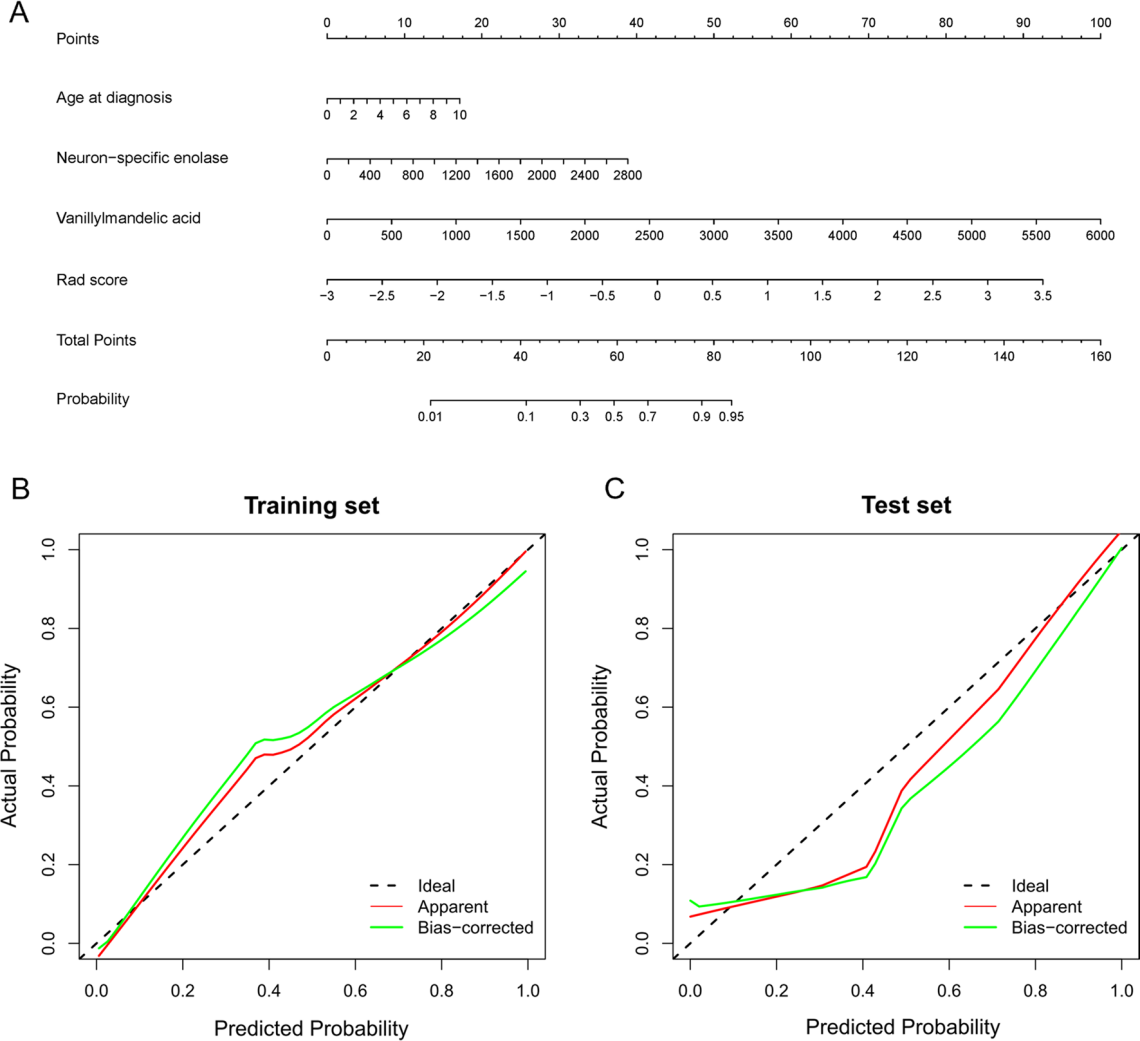
**A** Radiomics nomogram for non-invasively prediction of bone marrow involvement (BMI) in pediatric patients with neuroblastoma. The radiomics nomogram was a visual representation of the clinical-radiomics model in the training set, which incorporated the Rad score, age at diagnosis, neuron-specific enolase and vanillylmandelic acid. **B** Calibration curves of the nomogram in the training set. **C** Calibration curves of the nomogram in the test set

The Rad score, age at diagnosis, NSE and VMA were incorporated into the radiomics nomogram (Fig. 5A). The calibration curves demonstrated well consistency between the nomogram prediction and actual BMI in both the training and test sets (Fig. 5B, C). The radiomics nomogram achieved good predictive performance with AUCs of 0.924 (95% CI: 0.850–0.968) in the training set and 0.925 (95% CI: 0.796–0.984) in the test set.

**Fig. 6:**
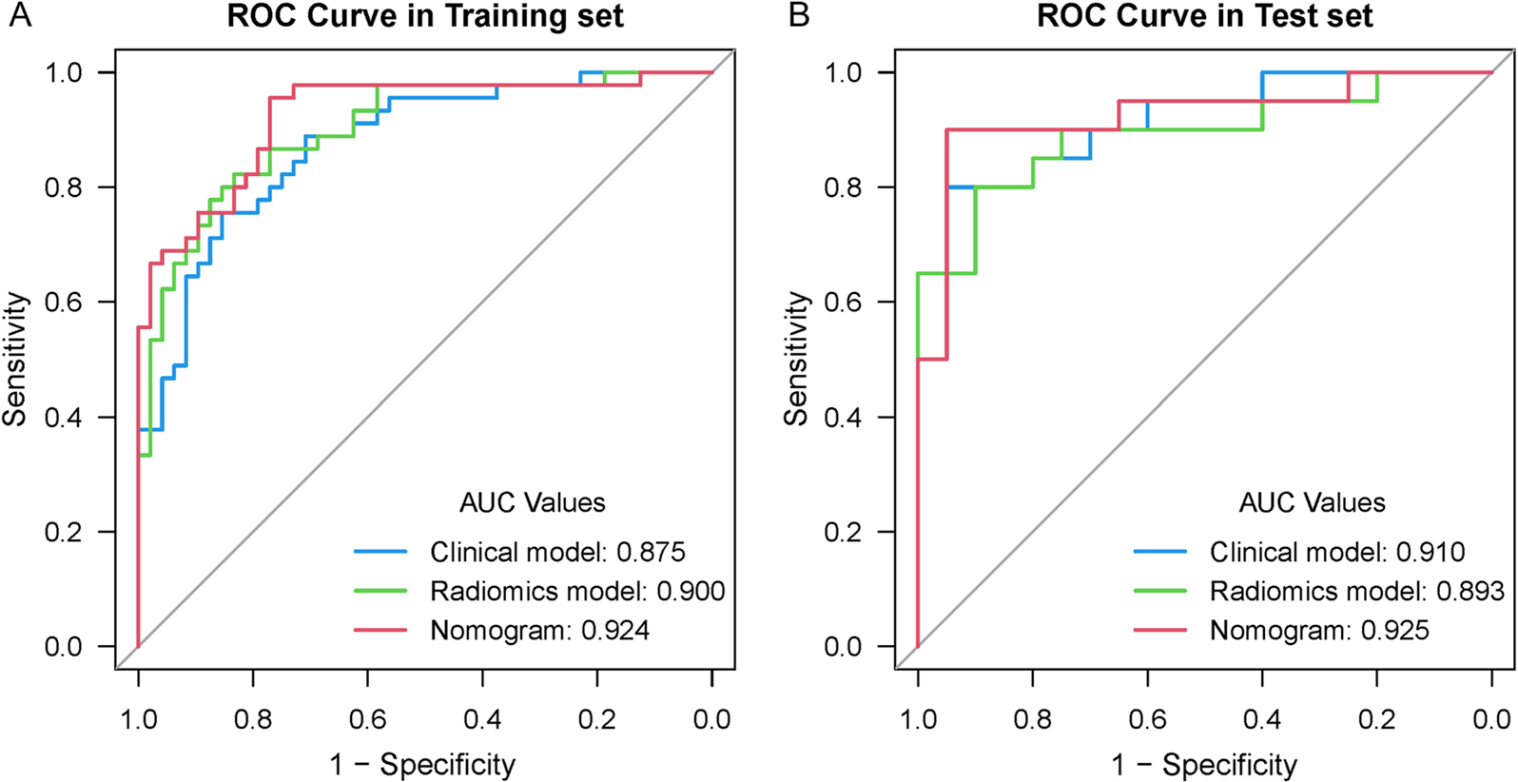
**A** Receiver operating characteristic (ROC) curves for the clinical model, radiomics model and nomogram in the training set. **B** ROC curves for the clinical model, radiomics model and nomogram in the test set

**Fig. 7:**
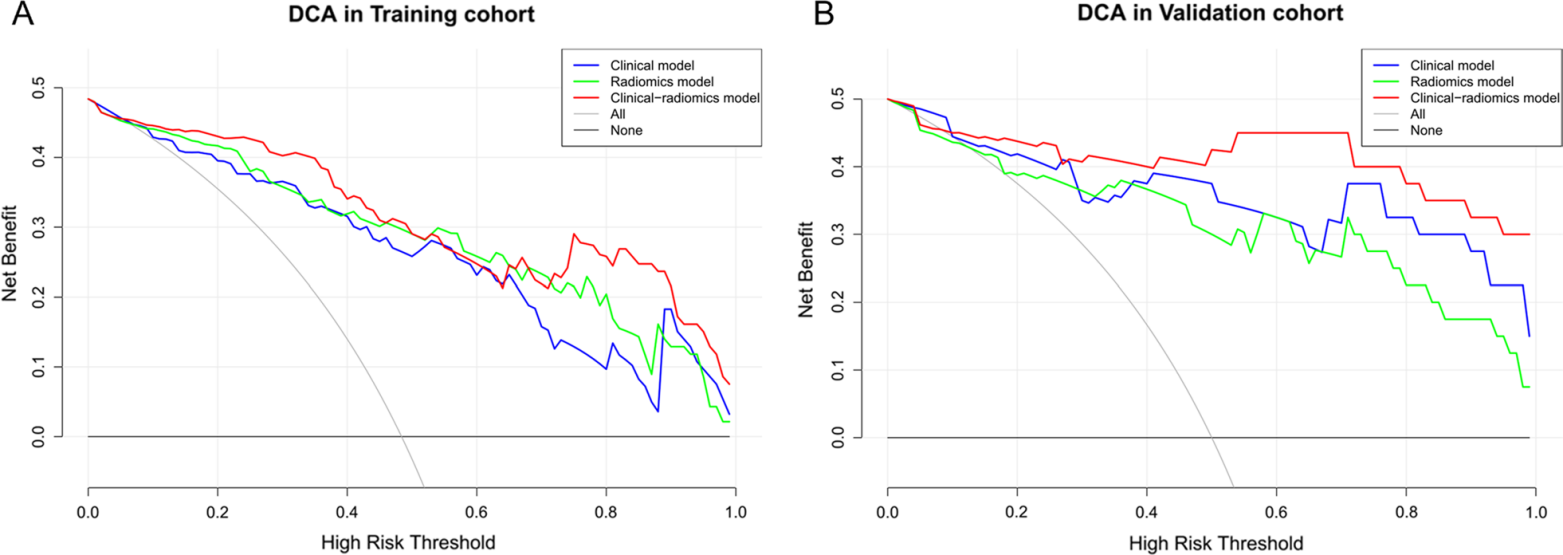
**A** Decision curve analysis (DCA) for the clinical model, radiomics model and nomogram in the training set. **B** DCA for the clinical model, radiomics model and nomogram in the test set

Figure 6 showed the comparison of ROC performance between the radiomics models, clinical model and clinical-radiomics model in the training set and the test set. In the training set, the radiomics nomogram outperformed the radiomics model or clinical model (AUC: 0.924 vs. 0.900, 0.875; *p* = 0.160, 0.035), which was then confirmed in the test set (AUC: 0.925 vs. 0.893, 0.910; *p* = 0.349, 0.732). The DCA for the different models in the training and test sets were shown in Fig. 7. It demonstrated that the radiomics nomogram resulted in higher overall net benefits than either the radiomics model or the clinical model alone.

The results of the predictive performance of the clinical-radiomics model by the different machine learning methods in the training and test sets are described in the Additional file (Additional file 2: Table 1).

## Discussion

Initial evaluation of BMI in patients with neuroblastoma had a crucial influence on the patient’s prognosis, clinical decision-making and further management. In the present study, we constructed an ^18^F-FDG PET/CT-based radiomics nomogram for the first time as a non-invasive method to predict BMI in pediatric patients with neuroblastoma. The radiomics nomogram incorporating the age at diagnosis, NSE, VMA and Rad score had an excellent predictive performance in both the training and test sets, with an AUC of 0.924 and 0.925, respectively.

Cytology of aspirates and histology of biopsies have been the gold standard with which to assess neuroblastoma disease in bone marrow for many years [6]. However, bone marrow aspirates and biopsies have a significant risk for pediatric patients that entails a multistep process with technical challenges and diagnostic complexity [27]. Radiomics is an emerging field, that has developed rapidly in recent years with the development of precision medicine [28]. Radiomics uses many automated data characterization algorithms to convert images of the ROI into quantitative high-throughput features, which radiologists cannot do with the naked eye [29]. By analyzing and calculating the quantitative features extracted from medical images to reflect information about tumor biology and microenvironment, it can elaborate on intra-tumor heterogeneity more effectively and accurately. It has become a useful imaging marker and can non-invasively provide the information for the diagnosis, improve the differential diagnosis, the classification of risk and staging, efficacy assessment, and prognosis of tumors [30]. ^18^F-FDG PET/CT has the advantages of superior spatial resolution, high sensitivity, lesion semi-quantification, improved tumor-to-background contrast, and is widely used in the evaluation of pediatric neuroblastoma and metastasis [31]. A study compared different imaging modalities in 51 patients with high-risk neuroblastoma and concluded that ^18^F-FDG PET/CT is highly effective in identifying neuroblastoma for revealing small lesions, and for delineating the extent and localizing sites of disease [32]. In our study, a total of 2632 radiomics features were extracted from both CT and PET images. Finally, twenty-five radiomics features based on the ^18^F-FDG PET/CT were selected by LASSO regression. The twenty-five radiomics features were used to construct the radiomics model and demonstrated a favorable ability to predict BMI in both the training and test sets. The radiomics model achieved an AUC of 0.900 in the training set and 0.893 in the test set in predicting BMI in children with neuroblastoma. Of the twenty-five radiomics features, the majority of selected features were wavelet features (18/25). It was indicated that features extracted from the images transformed by wavelet filter played an important role in the radiomics model. The wavelet filter can decompose special patterns hidden in the mass of data and the wavelet features may better explore tumor heterogeneity [21].

In addition to radiomics analysis, we also evaluated the value of clinical characteristics in predicting BMI. The age at diagnosis, NSE and VMA were identified as the independent predictors of BMI by univariate and multivariate logistic regression analyses. The children with age at diagnosis ≥ 18 months had a significantly higher risk of recurrence than those with age < 18 months [33]. And for patients aged ≥ 18 months at diagnosis, there is a clear correlation between worse outcomes with the increasing metastatic burden (including BMI) [4]. NSE, a specific isoenzyme of the glycolytic enzyme enolase, is highly expressed in neurons and peripheral neuroendocrine cells. Previous studies indicated that serum NSE levels ≥ 100 ng/mL were associated with a poor outcome [34]. Neuroblastoma can synthesize and secrete catecholamines and VMA is increased in the urine of children with neuroblastoma. Thus, the serum levels of NSE and urine levels of VMA are considered characteristic tumor markers of neuroblastoma [33], which can assess the condition, predict the effect of treatment and evaluate the prognosis of children with neuroblastoma [34].

Due to heterogeneity of clinical presentation of neuroblastoma, this disease is divided into clinically distinct subgroups, high, intermediate, and low-risk groups, based on different parameters, including the extent of the disease (whether or not with BMI). According to the INSS, neuroblastoma was staged as stages 1, 2, 3, 4 and 4S. Tumors with dissemination to distant lymph nodes, bone, bone marrow, liver, skin and other organs were defined as stage 4. Of the stage 4 neuroblastoma patients, 78% have BMI. The spread of tumor cells to bone marrow is a grim prognostic factor in neuroblastoma patients, with a low 5-year event-free survival rate (29%) [4]. Therefore, BMI, age at diagnosis, NSE and VMA are all associated with poor prognosis in neuroblastoma. The present study demonstrated that the age at diagnosis, NSE and VMA as independent predictors of BMI. The clinical model included the three independent predictors with an AUC of 0.875 and 0.910 in the training and test sets, respectively.

Furthermore, we developed and validated an ^18^F-FDG PET/CT-based radiomics nomogram that incorporates the Rad score and independent predictors of BMI, which showed a preferable prediction of BMI in pediatric patients with neuroblastoma. Comparisons and evaluations of each model by DCA further demonstrated that the radiomics nomogram resulted in net benefits of providing more than the clinical model or radiomics model within a certain threshold probability range. The results showed that radiomics nomogram may provide the physician with an independent or auxiliary predictive tool to enhance the diagnostic efficiency for BMI in patients with neuroblastoma.

There were some limitations in this study. Our study was retrospective, which inevitably result in selection bias. Moreover, this was a single-center study and the sample size was relatively small, which makes it less generalizable to other centers, therefore, the clinical application and generalization of the model still need to be further improved and validated by multicenter studies with a larger sample size.

## Conclusions

In conclusion, the ^18^F-FDG PET/CT-based radiomics nomogram which incorporates Rad score and independent clinical risk factors (including the age at diagnosis, NSE, and VMA) showed satisfactory value for the prediction of the BMI in pediatric patients with neuroblastoma in this preliminary study, and larger series are required to confirm this study’s results. As a non-invasive quantitative method, it holds the potential to assess the prognosis of the patients and assist in decision-making and further management of neuroblastoma.

## Supplementary Information

**Additional file 1**. Complete dataset.

**Additional file 2**. **Supplementary Fig. 1** The selected twenty-five features and their coefficients, and the formula for the Rad score. **Supplementary Table 1** The performance of the clinical-radiomics model by the different machine learning methods in the training and test sets.

## Data Availability

The datasets analyzed during the current study are available from the corresponding author on reasonable request.

## Abbreviations

AUC: Area under the curve
BMI: Bone marrow involvement
CI: Confidence interval
DCA: Decision curve analysis
HVA: Homovanillic acid
ICC: Intraclass correlation coefficient
INSS: International Neuroblastoma Staging System
LASSO: Least absolute shrinkage and selection operator
LDH: Lactate dehydrogenase
MTV: Metabolic tumor volume
NSE: Neuron-specific enolase
OR: Odds Ratio
ROC: Receiver operating characteristic
ROI: Regions of interest
TLG: Total lesion glycolysis
VMA: Vanillylmandelic acid
