# Supplementary material for: 18F-FDG PET/CT-based radiomics nomogram could predict bone marrow involvement in pediatric neuroblastoma
Source: Insights Imaging. 2022 Sep 4;13:144. doi: 10.1186/s13244-022-01283-8 (PMC9440965; doi:10.1186/s13244-022-01283-8)
Supplement: Supplementary file 2 — Additional file 2. Supplementary Fig. 1 The selected twenty-five features and their coefficients, and the formula for the Rad score. Supplementary Table 1 The performance of the clinical-radiomics model by the different machine learning methods in the training and test sets. [file 13244_2022_1283_MOESM2_ESM.docx]

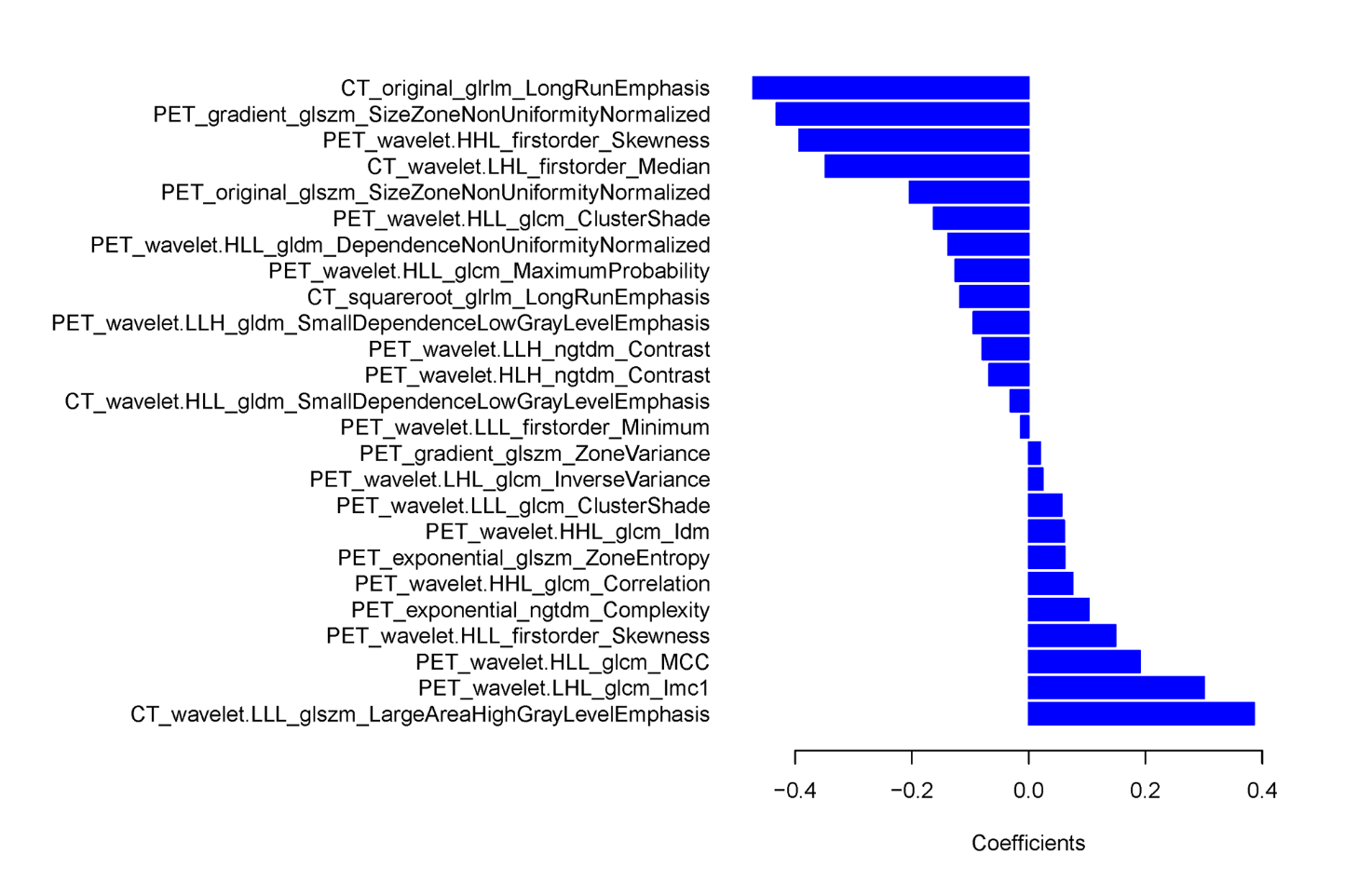


**Supplementary Fig.1** The selected twenty-five features and their coefficients.

The Rad score was calculated as the following formula:

Rad score = -0.05931760 -0.47162014× CT_original_glrlm_LongRunEmphasis

- 0.11733233× CT_squareroot_glrlm_LongRunEmphasis

- 0.34771009 × CT_wavelet-LHL_firstorder_Median

- 0.03104470 × CT_wavelet-HLL_gldm_SmallDependenceLowGrayLevelEmphasis

+ 0.38610957× CT_wavelet-LLL_glszm_LargeAreaHighGrayLevelEmphasis

- 0.20416190 × PET_original_glszm_SizeZoneNonUniformityNormalized

+ 0.06161197 × PET_exponential_glszm_ZoneEntropy

+ 0.10321253× PET_exponential_ngtdm_Complexity

-0.43179518 × PET_gradient_glszm_SizeZoneNonUniformityNormalized

+ 0.01990588× PET_gradient_glszm_ZoneVariance

- 0.09466926 × PET_wavelet-LLH_gldm_SmallDependenceLowGrayLevelEmphasis

- 0.07915366 × PET_wavelet-LLH_ngtdm_Contrast

+ 0.30045160 × PET_wavelet-LHL_glcm_Imc1

+0.02458552× PET_wavelet-LHL_glcm_InverseVariance

+ 0.14853671× PET_wavelet-HLL_firstorder_Skewness

- 0.16229988 × PET_wavelet-HLL_glcm_ClusterShade

+ 0.19074104 × PET_wavelet-HLL_glcm_MCC

- 0.12567077 × PET_wavelet-HLL_glcm_MaximumProbability

- 0.13780665 × PET_wavelet-HLL_gldm_DependenceNonUniformityNormalized

- 0.06759616× PET_wavelet-HLH_ngtdm_Contrast

- 0.39298943× PET_wavelet-HHL_firstorder_Skewness

+ 0.07554402 × PET_wavelet-HHL_glcm_Correlation

+ 0.06077745 × PET_wavelet-HHL_glcm_Idm

- 0.01315503 × PET_wavelet-LLL_firstorder_Minimum

+ 0.05663641 × PET_wavelet-LLL_glcm_ClusterShade

| **Supplementary Table 1** The performance of the clinical-radiomics model by the different machine learning methods in the training and test sets | | | | | | |
| --- | --- | --- | --- | --- | --- | --- |
| Set | Machine learning methods | AUC  (95%CI) | Recall  (95%CI) | Accuracy (95%CI) | Precision | F1-score |
| Training | logistic regression | 0.924  (0.869-0.978) | 0.956  (0.849-0.995) | 0.860  (0.773-0.923) | 0.796 | 0.869 |
|  | naive bayes | 0.882  (0.810-0.954) | 0.933  (0.817-0.986) | 0.849  (0.760-0.915) | 0.792 | 0.857 |
|  | neural network | 0.903  (0.845-0.960) | 0.911  (0.788-0.975) | 0.860  (0.773-0.923) | 0.820 | 0.863 |
| Test | logistic regression | 0.925  (0.835-1.000) | 0.900  (0.683-0.988) | 0.925  (0.796-0.984) | 0.947 | 0.923 |
|  | naive bayes | 0.893  (0.779-1.000) | 0.800  (0.563-0.943) | 0.875  (0.732-0.958) | 0.941 | 0.865 |
|  | neural network | 0.881  (0.782-0.981) | 0.850  (0.621-0.968) | 0.825  (0.672-0.927) | 0.810 | 0.829 |
| AUC: area under the curve; CI: confidence interval | | | | | | |
